# The State of Animal-Assisted Interventions: Addressing the Contemporary Issues That Will Shape the Future

**DOI:** 10.3390/ijerph16203997

**Published:** 2019-10-18

**Authors:** Aubrey H. Fine, Alan M. Beck, Zenithson Ng

**Affiliations:** 1 California State Polytechnic University, Pomona- Department of Education 3801 W Temple Ave, Pomona, CA 91768, USA; 2 Center for Human-Animal Bond, College Veterinary Medicine, Purdue University, West Lafayette, IN 47907, USA; abeck@purdue.edu; 3 University of Tennessee College of Veterinary Medicine, 2407 River Drive, Knoxville, TN 37996, USA; zng@utk.edu

**Keywords:** Animal-Assisted Interventions, animal welfare, professionalization, public policy, human animal interactions

## Abstract

As the worldwide popularity of animal-assisted interventions (AAIs) increases, the field is quickly approaching a paradigm shift, adjusting its image to incorporate more evidence-based research and aligning its purpose for advancing a new future. Contemporary critical issues that confront the field today include, but are not limited, to research, animal welfare, practice guidelines, and public policy. This article will provide an overview of the history of AAI and the major milestones that the field has undergone. The current state of AAI research will be scrutinized, and the areas that warrant further study will be recommended. Special attention will be given to the current state of animal welfare in AAI, the research that has been done in the area, and practice guidelines that safeguard animal wellbeing. This article will then discuss how evidence-based research and animal welfare guidelines inform the development of comprehensive professional standards and influence changes in public policy regarding AAI. The authors’ perceptions for the field’s future trajectory will be presented, which will include solutions to move the field in the direction that best advances the human-animal bond in research, practice, and public perception.

## 1. Introduction

The field of human-animal interactions (HAI) and, more specifically, animal-assisted interventions (AAI) has greatly evolved over the past half century. Our association with animal companions and health has a long history. Specifically, the field of AAI is becoming a more recognized form of complementary therapy [1]. Both areas of investigation and practice have evolved from mainly misunderstood and sensationalized relationships between humans and animals, emerging as legitimate fields of study and service. What was once thought as somewhat novel and unusual is now generating more enthusiasm not only by the public, but also by growing numbers of interdisciplinary scientists and practitioners interested in studying and applying HAI. Many, including these authors, believe that the field of AAI is quickly approaching a paradigm shift, adjusting its image to incorporate more evidence-based research and aligning its purpose for the new future.

Nevertheless, Fine argued that perhaps the most significant misunderstanding of HAI has been the misrepresentation and possible exaggeration of AAI’s impact on the humans served [2]. The overemphasis on outcomes that is more anecdotal than evidence-based may have slanted some peoples’ perceptions. Furthermore, some of the misunderstanding may be attributed to the media’s excessive and unscientific focus on the relationship between animals and humans, creating an impression that a simple pet prescription is all that’s needed for miracles to occur [2]. Fine argued that the leadership in the field must help educate stakeholders about the value of these interactions and provide a realistic presentation that does not exaggerate the impact of these interactions. As Fine states, “Animal-assisted interventions should not be considered as a panacea, but should be considered as a valuable life opportunity that can make a difference” [2]. Scientists and practitioners must be more tempered and realistic in their explanations of the efficacy of AAI, especially since the scientific evidence is still not strong enough to support such high convictions. 

In this article, the authors will focus on the challenges that continue to plague the field, as well as provide some insights for solutions and directions for the future. Some of the topics discussed will focus on the state of research in the field, the importance of animal welfare, the need for more refined professional standards (e.g., standardizing definitions, training, certification), and the importance of shifting and supporting public policy changes in order to impact the future of the field.

## 2. A Glimpse at Our History 

“A small pet animal is often an excellent companion for the sick, for long chronic cases especially. A pet bird in a cage is sometimes the only pleasure of an invalid confined for years to the same room. If he can feed and clean the animal himself, he ought always to be encouraged to do so” [3].

Even before Florence Nightingale (1869) used animals in a therapeutic setting, the Quaker York Retreat in England, the first recorded use of animals in a therapeutic setting in 1792, utilized rabbits and poultry [4]. In the early 19th century, groups were beginning to train dogs to assist blind people in navigating their world [5].

Fine notes that the field of AAI has continued to evolve. The current landscape is quite different from its modern origin about 55 years ago [6]. It is hard to fathom that the early modern pioneers of AAI, such as Boris Levinson and Elizabeth and Samuel Corson, serendipitously discovered the therapeutic power of the human-animal connection. None of them anticipated what they witnessed. Although their findings were not intentional, the outcomes of their revolutionary animal-assisted therapy work impacted our future understanding of HAI. 

Fine reports that Levinson, a child psychologist practicing since the 1950s, noticed a child, who was typically nonverbal and severely withdrawn during treatment, began talking to and interacting with Levinson’s dog, Jingles, in an unplanned interaction [6]. This experience caused Levinson to see the possible benefits of utilizing a dog during his psychotherapy visits [7]. In 1964, Levinson coined the term “Pet Therapy.” Despite the anecdotal experience and valued outcome, Levinson initially resisted including his dog in therapy because he felt that incorporating Jingles would be considered too unorthodox. His initial beliefs were accurate. According to Levinson and Mallon, in his early lectures about his impressions of pet therapy, Levinson was ridiculed and belittled by his colleagues [8]. 

In the 1970s, Samuel Corson and Elizabeth O’Leary Corson were some of the first researchers to empirically study canine-assisted interventions at Ohio State University. Their findings revolved around what they initially witnessed with patients from Upham Hall Psychiatric Hospital when dogs were integrated into their daily programming [9]. The Corsons happened to have a group of dogs in a kennel nearby primarily used to study stress on dogs. Like Levinson, they inadvertently discovered that some of their patients with psychiatric disorders were interested in the dogs, including one patient who was also selectively mute. The Corsons discovered that interactions with the dogs made it easier for the patients to communicate with each other and the staff [10]. The Corsons coined the term “social lubricant” as one of the major outcomes occurring as a result of interactions between the client and a therapy animal. They observed the warmth generated in the HAI and believed it acted as a critical ingredient in forging a working relationship. Early experiences, such as those noted, opened the door to realizing that relationships with animals were not only beneficial for humans in their daily lives, but also could be valuable in therapeutic environments. This pioneer work encouraged the mental health community to consider the value of AAI. 

In the early years, there was little diversity in how AAI was implemented. In most cases, animals were included in therapy sessions to act primarily as social catalysts, and the outcomes reflected these expectations. Today, AAIs are viewed more robustly and are applied with diverse populations in a wide array of settings and purposes [1,11]. Furthermore, there is now a broad range of multidisciplinary professionals utilizing AAIs within their complementary human healthcare. Truly, the field is now at a crossroads, where science is catching up with what mainstream society has believed for years: our interactions with animals can positively influence human health. 

## 3. The Need for Research and Evidence: Connecting the Dots from Past to Present 

Initially, the field of HAI was supported primarily with rich anecdotal evidence. Although valuable as qualitative information, the field needed more substantial, evidence-based research that demonstrated its impact and efficacy in order to move the field forward and gain a more respected status. Today, the field is witnessing a new generation of scientists and practitioners who have picked up the torch and are leading the way towards responsible growth and exemplary service. 

By the 1970s, there was an awareness that companion animals could be used to alleviate human mental and physical health problems [10,12,13]. People were eager to see how their love of pets was not trivial but part of our social commitment to help those in need [14,15]. The broad-base support fostered a media happy to report positive stories about HAI and nurtured research studies using animals as therapeutic interventions for people in a variety of settings, creating a cultural confirmation bias [16]. Results have been varied owing to the difficulties associated with sample size, research design, and the difficulty to provide appropriate controls [17]. While studies in support of animal contact receive media attention, a very large longitudinal cancer study showed a shorter survival time compared to non-owners, especially for female cat or bird owners [18]. Another example is the media often touts dogs as relieving stress but one study found the impact is somewhat exaggerated [19]. Also, while there was never extensive research money, there was more funding for animal-assisted therapy (AAT) than for general animal studies of basic science value.

The earlier studies often utilized older adults, as the elderly were perceived to benefit the most due to having less social opportunity, starving for physical contact, and clustering that made the sample more accessible and numerous [20]. Although the majority of older people do not live in institutions, there were many studies using nursing home populations [21,22,23,24]. In 1987, the National Institutes of Health held a technology assessment workshop, announcing there are health benefits of animal companionship, and the summary report cover depicts an older woman with a cat on her lap [25].

Earlier studies relied on general observations and basic measures of change using interviews. Initially, researchers had to develop their own survey instruments; thus, there was no validation, and comparing studies was not easy [26,27,28]. More recently, qualitative information, like questionnaires, has been combined with quantitative analysis of data, often referred to as a mixed methods approach [29]. The earliest health issues addressed outside of the elderly population were focused on cardiovascular problems, which were prevalent and easy to assess, and the first AAI article to appear in a recognized peer-reviewed medical journal demonstrated that animal ownership improved the one-year survival rate after a cardiovascular event [30]. Over the years, there has been much literature in this area of study [31,32,33,34,35,36]. Now, there is an ever-growing interest to have animals facilitate activity, especially walking [37,38,39].

After the elderly, children were a common focus for AAI research in part because children are grouped together, but mostly because of the belief that children like animals and animals are good for them [7,8,40,41,42,43]. Today’s area of focus is autism [44,45,46], depression, trauma [47], and posttraumatic stress disorder (PTSD) [48], all emerging health and economic challenges to society. Regardless of the area of study, there is a need for establishing methods and standards for AAI studies [49,50,51]. Regarding research articles related to the human-animal bond, HAI, or human-animal relationships, both the rate of articles being published and the number of journals publishing these articles have steadily increased since the early 1980s [52].

AAI, like all scientific endeavors, is influenced by technology. There is an ever-growing reliance on methods perceived to be more valid and objective to assess the changes caused by animal interactions. Measurable physiological assessments, like oxytocin (looking for an increase) and cortisol (looking for a decrease), are becoming almost standard methods to assess stress in both humans and therapeutic animals [53,54,55,56,57,58]. In addition, there are a growing number of studies that assess stress and other aspects of autism using facial recognition, which does not require touching the subject [59,60,61]. For real-time assessment, wristband monitors measuring blood pressure, activity, and stress are being deployed for both the human subject and animal intervention [47].

Data analysis also has developed beyond basic statistical analysis of small samples to larger multisite studies, along with systematic review and meta-analysis of existing studies [44,47,62,63,64,65,66,67,68,69]. Panel studies have been employed, studying the role of AAI in the aging community [24].

While some of the earliest uses of animals to improve the health of people were farm animals, modern studies most often use dogs, then companion horses [70,71,72], and, to a lesser extent, cats [43,73]. Within the last three decades, other species have been used, including fish [23,62,74,75], birds [76], guinea pigs [47], and reptiles [77]. There is now growing literature on the use of robots that resemble and behave like animals [78,79,80,81].

The use of animals is now appearing in therapeutic settings, such as emergency departments [82], acute and hospice care [83,84], and elementary schools [42,85]. An ever-expanding area is the use of animals to prevent or diagnose health issues, such as detecting pathogens in the environment [86,87] or diagnosing cancers [88,89,90].

There is also a growing appreciation for the ethical issues surrounding AAI, including the potential health risks associated with animal contact [91], inappropriate animal ownership [92,93], and the misuse of animal assistance [94]. We see not only passive concerns for the welfare of animals used in therapy, but also dedicated studies to objectively assess the stress and general welfare of animals used [95,96,97]. The limitations of AAI must be recognized, so it can truly be for those people and animals that can benefit and objectively assess the science and public health value of the programs [14,98,99]. Most importantly, scholars must continue investigating the theoretical bases of AAI/HAI for the benefits of animals and nature in general [100,101,102]. This approach will improve the methods available for AAI and the welfare and benefits for the animals [103].

## 4. Advances in AAI must Consider Animal Welfare

Simply put, AAI would not exist without animals. Therefore, it is critical that we ensure the health and welfare of these beings in all aspects of AAI. Because the purpose of AAI is to use an animal to directly benefit a human, it is of utmost importance that the animal is not negatively impacted. In the history of human-animal relationships, animals have largely been viewed from a utilitarian point of view. The question has changed from “what can we do to our animals” to “what can we do for our animals.” While benefits to humans utilizing AAI are becoming more lucid, the benefits and consequences to the animals are not always clear or measurable. As the research begins to create a stronger evidence platform concerning the efficacy of AAI, Tedeschi questions if the field is ethically prepared to investigate the potential pressures on animals integrated into the interventions [104]. A chasm exists between what human clinicians are trying to explore in working with their clients and a thorough understanding of the risks the animals may face in regard to their services. People and AAI practitioners may assume these interventions are innocuous to the animals and that therapy animals typically enjoy the sessions. However, AAI poses a unique set of stresses and strains on animals that the field has recently begun to acknowledge [105]. The most discrete threats of AAI to animal welfare are the risk of zoonotic disease transmission from human to animal and the mental and physical stress a therapy animal may endure because of the work. 

Over the years, few studies have highlighted the potential stressors and challenges for animals participating in AAI [106]. Reports in the literature commenting on the moral basis of animal-assisted therapies suggest that posttraumatic stress disorder (AAA) and AAT exploit animals and may be detrimental to their well-being [107,108]. If AAI is not effective or beneficial for humans and potentially threatens the animal’s welfare, the justification for the use of animals for this purpose is questioned [107,108,109,110]. Fine discusses AAI in terms of a cost/benefit balance. The cost/benefit balance addresses the implications of AAI on the therapy animal’s quality of life [111,112]. To effectively safeguard the welfare of therapy animals, special considerations should be addressed before the animal enters therapy work, during therapy work, and after therapy work is complete.

### 4.1. Before Therapy Work

Perhaps the most important aspect of ensuring welfare of the therapy animal is choosing the right animal for the work. Most AAI organizations require the animal to pass a behavioral evaluation by a certified evaluator and a physical exam by a licensed veterinarian [113]. The use of a veterinarian knowledgeable about the skills required of and potential stressors to a therapy animal during the selection process cannot be underestimated [114]. This largely ensures the safety of the people interacting with the animal. The behavioral evaluation in dogs often constitutes a variety of tasks, including commands to sit, down, stay, come, and to walk on a loose lead. Additionally, evaluators often assess the animal’s reaction to strangers, other animals, medical equipment, loud and/or novel stimuli, angry voices, and/or potentially threatening gestures, crowds of people, being patted in a vigorous or clumsy manner, and being restrained in a hug [113,115]. The animal should be placed in role-play scenarios representative of a typical AAI encounter. The animal’s response to the AAI role-play should be predictable, and the ideal AAI animal should be friendly, confident, and composed. These traditional tests verify that the animal is non-aggressive during these challenges to assure human safety.

Although an animal may not demonstrate signs of aggression during a human-animal interaction, it does not necessarily mean the animal enjoys or is unstressed by the work. An obedient and trained animal may tolerate the interaction and behave accordingly but be internally distressed. In general, the animal should “enjoy” and seek interaction with strangers without showing signs of stress, fear, aggression, shyness, or avoidance [116]. The process of selecting therapy animals should focus on the subtle behavioral signs of stress that are often overlooked to ensure that the animal desires human interaction and will be successful in all environments [117]. Future research should validate the accuracy of these evaluations in predicting successful working careers. 

In the ideal practice of true AAT, the description of work and therapeutic goals should be specifically described and established beforehand, so an animal is selected only if it meets that job description [118]. This contrasts the typical practice of AAI, where the animal has already been screened and chosen simply because of availability. Even if an animal may not be suited for a specific job description, the globally registered therapy animal is molded to meet those goals. However, to best uphold animal welfare, the “goodness of fit” model should be utilized to appropriately mesh the animal’s temperament or personality with the demands [119]. This standard of practice requires premeditated thought, planning, and prioritization of animal welfare.

A significant influence of animal welfare in AAI that is not typically considered is the effect of the handler on the animal. The intimate dyadic relationship of the animal-handler team corroborates how the handler can impact the health and wellbeing of the animal. The handler is the gatekeeper of welfare and requires a minimum standard of education and skill to be safe and effective during AAI. Knowledgeable handlers must acquire an appropriate level of training in animal behavior and health. This training should include an emphasis on zoonotic disease; infection control practices; identifying appropriate contacts in the event of an accident or injury; reading an animal’s body language for signs of discomfort, stress, or fear; and patient confidentiality [115]. To assess the level of understanding from this training, the handler should successfully pass an assessment that ensures minimum competency in AAI. Pet Partners, a leader among AAI organizations, advocates the power of the handler by championing the phrase, “YAYABA,” an acronym for “You Are Your Animal’s Best Advocate” [113]. This illustrates a necessary commitment to assuring quality of interactions at both ends of the leash. Future research should investigate the effect of the handler’s skills and knowledge on the health, welfare, and performance of therapy animals.

### 4.2. During Therapy Work 

What is arguably the most intriguing aspect of animal welfare in AAI is the effect therapy sessions have on the animals. The effect of the therapeutic session on outcomes of various physiologic parameters (most commonly cortisol) and behavioral signs of stress has been the most frequently investigated aspect of animal welfare in AAI, although research in this area is still emerging. Multiple studies have reported that cortisol levels increased in dogs after AAI, [120,121,122,123] while others reported that cortisol levels did not change or decreased after AAI [96,97,124,125]. The increases in cortisol may, on the surface, be concerning since cortisol elevation is typically associated with negative distress. However, these elevations simply indicate that therapy work is physiologically arousing to dogs, but whether the arousal is due to positive excitement or distress requires a concurrent assessment of behavior or other physiologic measures. Many studies assessing behavior in therapy dogs have found no significant increases in stress behaviors during an AAI session compared to baseline [96,97,126,127]. 

Despite mixed results regarding the stress-related welfare concerns of working therapy dogs and a need for more research, the general practice of AAI does not appear to be overtly harmful to animals that are appropriately selected and responsibly handled. However, specific questions regarding their use, such as the ideal duration and frequency of interventions, have yet to be answered with scientific evidence. The reason these questions are difficult to answer with blanket recommendations is because no single AAI is identical, and each intervention will depend on the capacity of that particular animal engaging in that specific scenario. The stress an AAI may place on an animal is dependent on a number of factors, including the handler, the participant, the environment, and the interaction itself [128]. Future research should investigate the effect these factors have on animal welfare. Until more studies inform practice standards, it is up to skilled and knowledgeable handlers to uphold “YAYABA” and continuously monitor and assess the behavior of their animals and relieve animals from work before AAI becomes negatively impactful to their welfare. 

However, AAI should not be constantly viewed in a cynical light from an animal welfare perspective, as human interaction also benefits the animal. Human contact influences cardiovascular and hormonal outcomes that can be perceived as beneficial to the animal when the interaction consists of non-noxious sensory stimulation, including touch, light pressure, warmth, and stroking, as well as olfactory, auditory, and visual cues [129]. Human contact may also result in positive effects on endocrine function in animals, decreasing the activation of the hypothalamic–pituitary axis (HPA) and sympathetic nervous system [129,130]. 

The science surrounding the human-animal bond is particularly fascinating when it surpasses the question of exclusively positive or negative stress states and explores the complexity of how dynamic human-animal relationships influence cognition and emotion in the animal. Current research is increasingly recognizing that animals are sentient beings that have the ability to experience emotions [131]. A sentient being implies that the individual has the ability to experience emotional effects of pleasure and suffering. Animals may have the ability to be empathic, which is a quality that has yet to be scientifically characterized and may be key to how an animal interacts with a human to bring about therapeutic benefit. For example, a person that is emotionally distraught and crying may cause an animal to instinctively make physical contact with the person to comfort him or her. This becomes a welfare concern when it is perceived that the animal absorbs and is adversely affected by negative emotion. The ability to understand whether an animal has the cognitive ability to process emotions is unknown but may be elucidated with advances in technology and diagnostics to better understand cognition and wellbeing. Functional magnetic resonance imaging (fMRI) permits assessment of brain activity by detecting changes associated with blood flow in real time, demonstrating that different parts of the brain in dogs activate with different stimuli [132]. While this modality has the potential to demonstrate what truly goes on inside the head of a dog during AAI, its use is limited because the dog needs to be trained to enter the fMRI unit and remain still during the entire evaluative process [133].

However, other advances in diagnostics and better understanding the meaning of changes in various hormones make studying the effect of human interaction on the animal more attainable. Dogs that received positive human interaction not only experienced decreases in heart rate and blood pressure, but also significant increases in β-endorphin, prolactin, phenylethylamine, and dopamine levels associated with bonding, euphoria, pleasure, and happiness, suggesting positive effects of human interaction [125]. Oxytocin, often referred to as the “love” hormone because its increase is associated with positive interactions, such as maternal-infant bonding, friendships, marriage, and sex, has been of particular interest to study the positive emotional state in dogs after human contact [134]. In addition, the quantification of physiologic variables, such as heart rate variability (HRV) [135,136], immune status through neutrophil to lymphocyte ratios [137,138,139], acute phase proteins [138,140], and salivary immunoglobulin A, [141,142] may provide information regarding the stress state of the animal. The study of animal emotion, stress, and welfare is generally challenging, and requires simultaneous assessment of multiple physiologic and behavioral parameters. Future studies on the effect of AAI on animals should incorporate a variety of measures to obtain a comprehensive assessment of the animal.

The most assured recommendation to safeguarding welfare during the work is preventing the transmission of zoonotic disease by consistent hand washing [143]. There is evidence that therapy dogs have become colonized with *Clostridium dificile* (C diff), as well as methicillin-resistant Staphylococcus aureus (MRSA) [144,145,146], after visiting human healthcare settings. Although the animals were not clinically infected or ill, the potential for infection in the animal or the transmission to other individuals still exists. Since practices, such as shaking paws, getting up on beds, licking faces, and taking treats, were risk factors for colonization of MRSA and C diff, these activities should be avoided or kept to a minimum whenever possible. Hand washing is essential to the health of the animal and to all individuals who encounter that animal.

### 4.3. After Therapy Work

The welfare of the therapy animal after the AAI or the notion that therapy animals should “retire” from work is a topic that has received attention in the literature. Every being that has a job should be granted the reward of retirement from said job. From an animal welfare perspective, retirement is a critical and necessary phase of every therapy animal’s life. The AAI field should embrace the concept that that an animal may be relieved of duties if welfare is compromised during the work. Retirement is typically regarded as a well-deserved reward earned after a lifetime of work, but the termination of an animal’s career may also carry negative implications for animal, handler, and human participants in these interventions, particularly if retirement is not willingly chosen. An animal that thrives on human interaction may be frustrated when a change in physical health status warrants retirement. Another ethical conundrum presents itself when the therapy animal is ready to retire, but the handler and or the participants are not ready for the animal to retire. The ending of a therapy animal’s career is inevitable and can be challenging to navigate. Understanding the best time to retire and how to retire gracefully may minimize the consequences of ending this work. Future research should investigate the ideal time for retirement and how to appropriately retire an animal from work. 

### 4.4. The Future of Animal Welfare in AAI

While the field of AAI has turned attention to the welfare of the therapy animals, our current knowledge of the way AAI impacts the welfare of participating animals is still limited, with few studies rigorously evaluating its short- and long-term effects. Since AAIs are so variable and dependent on the individuals and circumstances, controlled prospective trials are scarce. Research utilizing innovative methods of animal welfare assessment and cognition should continue to inform the best practice standards to ensure the welfare of these animals. In addition, practice should elicit pertinent research questions by consistently reporting adverse outcomes and events from AAI. 

One important aspect of AAI to address is how researchers ensure the ethical treatment of the animals in AAI. Regardless of the research subject, any study that includes living beings warrants ethical review. While the Institutional Review Board (IRB) oversees the ethical treatment of humans in research, the Institutional Animal Care and Use Committee (IACUC), also termed as an animal ethics committee or animal welfare committee, oversees the ethical use of animals in research. It is the role of the IACUC to oversee an institution’s animal research programs, facilities, and procedures. IACUC oversight has been underutilized in AAI research [106], and the involvement of this committee is warranted to safeguard the use of animals in any study. Furthermore, the ability to replicate research methods is necessary for growth in the field, and most AAI manuscripts lack details to successfully reproduce a protocol. Minimum details that should be included in every AAI study are age, sex, species/breed, veterinary health status, training/certification, frequency, duration of intervention, and length of study. The incidence of adverse events, including infectious disease or injuries, should always be reported. Rigorous review and descriptions of animals in this type of research contributes to the quality and growth of the field of AAI.

Those engaged in AAI should be cognizant of the welfare of their therapy animals and incorporate practices supporting animal well-being. Fine and Mackintosh stress our moral responsibility to listen to therapy animals’ silent communications and make decisions that are in their best interest, placing animal welfare at a comparable level to patient outcomes [147]. Some therapy animals face challenges in their roles, both during their daily interactions or while preparing and returning from sessions. Additionally, other dilemmas may stem from various life situations, including declining health and retirement, leading to a decreased workload, modified interactions, or stopping work all together. In reaching these decisions, it is important to keep the wants and needs of the animal in mind. We must continually monitor the animals’ welfare and develop methods to measure fatigue and stress to achieve the best possible outcomes for both animals and humans. Globally instituted measures can help avoid pitfalls and safeguard the animals. The field has come a long way in asking not just what your animal can do for you, but what can you do for your animal.

## 5. Professionalization of AAI 

As the field continues to evolve and more disciplines become involved in the movement, there is a need for more clarity about the AAI spectrum and the requirements for practitioners to best implement these services. The International Association of Human–Animal Interaction Organizations (IAHAIO) published a white paper titled, “The IAHAIO Definitions for Animal Assisted Activity and Guidelines for Wellness of Animals Involved” in March 2013. Those appointed to serve on the formulation task force for the paper were academics, veterinary medicine professionals, and practitioners from different countries with a background in, or special knowledge of, varied dimensions in the field of HAI. The “Task Force was established and charged with the responsibility of clarifying and making recommendations on AAI and AAA terminologies and definitions and outlining ethical practices for the wellbeing of animals involved” [148]. It is the authors’ opinions that these are the most current definitions and should be utilized for the clarification of the definition of various aspects of AAI. 

Over the course of the past twenty years, we have witnessed not only more research on the efficacy of AAI, but also more specific research investigating the impact of various types of interventions with specific populations. For example, O’Haire et al. prepared a systematic review of animal assisted interventions and their impact in working with persons that experienced trauma [47]. The team identified several studies that demonstrate the efficacy of applying these principles with children who are victims of abuse and some preliminary research on understanding how AAI can be valuable with war veterans. Furthermore, O’Haire also prepared similar papers on demonstrating the value of AAI with persons with Autism [44,149]. She provided insights on how AAI could be applied effectively with persons with ASD. Gabriels et al. and Grandin et al. also provided insight into how equine assisted interventions can be implemented effectively with this population [150,151]. Finally, Schuck et al. provide a comprehensive discussion on how AAI can provide support for children who have executive functioning disorders, specifically ADHD [152,153,154]. The unique aspect of Schuck’s work was the development and application of a specific protocol incorporating canine assisted interventions. This protocol has been replicated several times with similar outcomes. One of the recommendations from O’Haire’s work was the need to provide detailed information with regard to procedures so that independent research teams could continue to validate the efficacy of protocols [47]. She strongly suggested publishing manuals for more widespread use. This is now being done by more practitioners to try to disseminate the work being incorporated. For example, Schuck is working on completing the PACK manual, which details the procedures and the training necessary to incorporate AAI with children with ADHD in clinical settings. 

As AAI continues to evolve, more specificity in its application in various environments and with diverse populations continues to evolve. Fine reports about the diverse setting, where AAI continues to expand from more accepted roles (i.e., hospital-based settings, nursing homes, and schools) to roles as stress buffers in forensic courtrooms and disaster areas [1]. All of these new expansions will need further research and evidence to clarify how they can best be practiced. However, the findings of the research in many of these areas has clearly identified the value of AAI with these various populations. For example, Fine and Friedmann have noted that much of the research on the contribution of human-animal interactions to social interaction in older adults is derived from studies of animal assisted interventions for individuals with cognitive impairment who reside in long-term care facilities [155]. A synthesis of the findings from the research seems to indicate that AAI improved both the quantity and the quality of social interaction in the elderly population [156]. Furthermore, several studies with older adults demonstrated that human-animal interactions lead to decreases in depression among psychiatric patients, individuals with disabilities or individuals who live in assisted living facilities [157,158,159,160]. Additionally, findings from research also pointed out that pet ownership tended to be associated with survival of seniors living independently in their community who had experienced a heart attack. These individuals also appeared to have fewer symptoms of depression [159]. 

On the other hand, over the past fifteen years there has been strong interest in studying the value of including animals in educational settings [161]. Currently, several studies have been initiated investigating the impact of incorporating various species of animals in supporting the cognition, social competence and motor development of the children. Findings from these studies have been very promising [156,162,163,164,165,166,167]. Furthermore, an area that has received a great deal of interest pertains to the impact that dogs have in supporting students in reading. Hall et al initiated a systematic review on this topic and examined 27 studies specifically on teaching benefits of children reading to dogs [168]. The researchers suggested that there is positive evidence indicating the value of reading to a dog. They believe that one of the strongest benefits is derived from the interaction between the dog and the child. It appears that the dog has an impact on the child’s mood elevation. Furthermore, in their review of the literature, the authors also suggested that the relationship between the dog and the child appears to have an impact on the child’s arousal level while reading. In essence, children that are comfortable with the dogs and have positive interactions, appear to have a decrease in their anxiety in reading to another. 

Looking into the future, our shared vision must promote the professionalization of the field of AAI in becoming a more recognized form of complementary medicine in order to gain the stature of being a valued treatment modality. The National Center for Complementary and Alternative Medicine (NCCAM), one of 27 institutes of the NIH in the United States, defines complementary medicine as “using a non-mainstream approach together with conventional medicine” [169]. AAI fits well within this definition, as it is typically incorporated with traditional forms of therapy, such as psychotherapy and occupational and physical therapy. 

Fine et al. report that continuing education and certification of competencies may very well be the key in taking AAI to the next level of field-focused growth, especially in terms of building credibility and moving toward acceptance as a complementary therapy [170]. We believe that the future will require specific and mandated education and training to ensure effective, safe, and reliable treatment options. Academia’s role is critical in becoming a clearinghouse for information, education, and research applicable to various professions.

## 6. Public Policy: Changing One Mind at a Time 

According to McCune et al., scientific research has always been used to better understand the world around us and to help us validate or demystify our theories and preconceived notions [103]. It is only logical that research will be used to advance the field of AAI and answer many questions that are posed by the field. Feldman et al. argue that society needs science to document the positive benefits of human-animal interaction in order to make changes to present-day policies [171]. Policymakers, health care professionals, and other decision-makers need scientific data to advocate more positively for the value of HAI. When such information is made available, the gatekeepers of public policy and resources will be more receptive to open up new opportunities for AAI. 

Although we have made great strides in public awareness of the value of co-existing with animals, according to Arkow, bureaucrats seem to be more interested in specific outcomes that demonstrate tangible benefits for humans [172]. Arkow, in a presentation entitled “Learn what the monster likes and feed it,” stresses that officials in health organizations, education, and various levels of government do not seem to be primarily influenced by specific animal welfare issues [173]. Rather, they appear responsive to outcomes where animals in our communities, homes, and therapy make a difference to people’s health, safety, and economics. We strongly urge the research community to develop a platform for public policy that bridges research with practice, supporting the value of AAI. These translational research efforts that bridge both research and practice will inform and drive public policy. While more scientifically based evidence will always be needed to convince skeptics, the broader societal trajectory for AAI suggests that the movement from research to practice is accelerating. The challenge then is to attempt to document needed best practice information for practitioners, and to support those practices with enlightened public policies, even as vital research continues. For example, more attention is needed to understand best practice approaches, which include the specific type of intervention and how much time and intensity is needed to find a change (dosage questions). Feldman et al. conclude by suggesting that research can help determine how to impact the greatest number of people in need and implement interactions already utilized in current practice [170]. Research can drive practice, and practice can drive research. When research and practice complement each other, we can drive public policy change, especially when the information pipeline is established and key parties are constantly informed.

## 7. Conclusions

In 2005, Steve Jobs, the founder of Apple, gave a commencement speech at Stanford University. It was perhaps his most prolific speech, as he encouraged new graduates to aspire to be their best. In his speech, he notes, “you cannot connect the dots looking forward; you can only connect them looking backwards, so you have to trust that the dots will somehow connect in your future” [174]. The dots in AAI are continuing to connect. The field began with leadership who became infatuated with what they witnessed. Levinson and the Corsons observed first-hand the impact of patient interactions with warm-hearted dogs. Their anecdotal findings and early efforts inspired generations of researchers and clinicians to study and develop more reliable and effective interventions incorporating therapeutically designed HAI. 

The field’s landscape over 65 years later looks very different but still possesses some of the same passion for unearthing the power within human-animal connections. We are beginning to understand what some have intuitively believed for centuries: having animals in our lives can be good for most of us. Connecting the dots backwards allows us to see how these serendipitous findings began a movement the early pioneers would be proud to endorse. There is a paradigm shift as the field continues to evolve that will help solidify and clarify the benefits of AAI. This shift is taking place because of the efforts of both the scientific community and those engaged in clinical practice. With the help of researchers, the field continues to flourish by studying not only the impact of these interactions, but also the biological mechanisms that are changing in both humans and their animal counterparts. The better-trained group of interdisciplinary professionals continues to develop more evidence-based interventions, underscoring the efficacy of this shift in approach. The ultimate beneficiaries are both clients and animals. The field will benefit most by focusing on the objectives to use evidence-based outcomes to inform practice standards; to apply techniques that ensure animal wellbeing; to share universal resources, terminology, and practice standards; to advocate AAI in public policy; and to provide formal training and education that promotes professionalization of the field.

As Steve Jobs elegantly noted, you have to trust that the dots will connect sometime in the future. The AAI field will continue our efforts to connect the dots towards the future by developing a research and practice platform that continues to investigate the impact of human-animal connections.
